# Unsupervised Deep Representation Learning and Probabilistic Clustering for the Systems-Level Discovery of Germline Mutation Signatures in Pediatric Cancers

**DOI:** 10.3390/biomedicines14071438

**Published:** 2026-06-24

**Authors:** Fahimeh Palizban, Michael E. March, Xiang Wang, James Snyder, Fengxiang Wang, Frank Mentch, Yeshwanth Mahesh, Alexandria Thomas, Deborah J. Watson, Huiqi Qu, John Connolly, Amir Hossein Saeidian, Hassan Vahidnezhad, Joseph Glessner, Hakon Hakonarson

**Affiliations:** 1 Center for Applied Genomics CAG, The Children’s Hospital of Philadelphia, Philadelphia, PA 19104, USA; palizbanf@chop.edu (F.P.); quh@chop.edu (H.Q.);; 2 Department of Bioengineering, University of Pennsylvania, Philadelphia, PA 19104, USA; 3 Department of Pediatrics, Perelman School of Medicine, University of Pennsylvania, Philadelphia, PA 19104, USA; 4 Department of Molecular and Human Genetics, Baylor College of Medicine, Houston, TX 77030, USA; 5 Division of Human Genetics, Children′s Hospital of Philadelphia, Philadelphia, PA 19104, USA; 6 Department of Dermatology, Perelman School of Medicine, University of Pennsylvania, Philadelphia, PA 19104, USA; 7 Division of Pulmonary Medicine, The Children’s Hospital of Philadelphia, Philadelphia, PA 19104, USA

**Keywords:** pediatric cancer, germline mutation signatures, unsupervised machine learning, deep learning, retrospective cohort study, systems genomics, precision oncology

## Abstract

**Background/Aims**: While pathogenic germline variants play a critical role in pediatric cancer susceptibility, traditional clinical genetics primarily focuses on single-gene interpretations. Transitioning to a systems-level analysis of inherited variation can uncover shared biological vulnerabilities, informing genetic counseling, surveillance, and targeted therapeutics. This study aims to implement an unsupervised machine learning framework to identify and characterize Germline Mutation Signatures (GMS) across diverse pediatric malignancies, elucidating latent genomic patterns that reveal shared oncogenic mechanisms. **Methods**: We analyzed germline whole-exome and whole-genome sequencing (WES/WGS) data from a retrospective cohort of 420 pediatric cancer patients and matched non-cancer controls. Variants were deeply annotated to capture multi-dimensional features, including predicted pathogenicity, splice-site disruption, regulatory impact, population frequency, and sequence context. To enable robust modeling, we integrated an augmented feature set encompassing evolutionary constraint, loss-of-function intolerance, and compositionally normalized substitution spectra. These high-dimensional annotations were processed using a deep autoencoder for non-linear representation learning, followed by Gaussian Mixture Modeling (GMM) of the latent space. **Results**: The framework delineated 13 signatures (GMS1–GMS13), yielding an optimal Davies–Bouldin index of 1.051. These signatures map to fundamental biological processes, including DNA repair deficiencies, transcription-coupled damage, replication stress, and aberrant RNA regulation. Crucially, these GMSs transcend traditional tissue-of-origin classifications, manifesting across multiple distinct cancer types. This observation indicates convergent germline etiologies and suggests potential shared susceptibilities to pathway-directed therapies. **Conclusions**: The discovery of these cross-cancer signatures provides a scalable, biologically interpretable framework for decoding inherited pediatric cancer risk. While the therapeutic mapping networks identified are currently exploratory and serve as a hypothesis-generating foundation, this deep learning-driven paradigm establishes a robust basis for stratified precision medicine. Pending prospective clinical validation, this approach holds significant translational potential to move beyond single-gene paradigms toward unified, systems-level precision oncology strategies.

## 1. Introduction

Pediatric cancers, though relatively rare compared to adult malignancies, remain a leading cause of disease-related mortality among children and adolescents worldwide, with an estimated 400,000 new cases diagnosed globally each year [1]. Unlike adult cancers, which are predominantly driven by the progressive accumulation of somatic mutations due to environmental exposures and aging, pediatric oncogenesis is fundamentally rooted in developmental biology [2]. Recent large-scale sequencing initiatives have demonstrated that approximately 8% to 15% of pediatric cancer patients harbor pathogenic germline variants in known predisposition genes, a prevalence that significantly exceeds that observed in many adult cohorts [3]. From a clinical and healthcare perspective, the role of germline variation in pediatrics is three-fold: (1) it serves as the primary driver of oncogenesis during critical developmental windows; (2) inherited defects in DNA repair and replication fidelity shape the tumor’s mutational landscape and subsequent relapse patterns; and (3) these variants directly influence treatment toxicity and pharmacogenetic response, making their detection vital for long-term survivorship [4]. Despite these critical implications, traditional clinical genetics remains largely gene-centric, focusing on the interpretation of single variants while overlooking the cohort-level patterns that could reveal shared systems-level risk. This focus on individual Mendelian risk factors creates a significant gap in healthcare delivery, as it often fails to provide context for the high volume of Variants of Uncertain Significance (VUS) or identify pan-diagnostic biological vulnerabilities that transcend traditional tissue-of-origin classifications [5]. Transitioning to a systems-level analysis of inherited variation specifically through the discovery of Germline Mutation Signatures (GMS) offers a promising framework for advancing precision oncology. By summarizing genome-wide features into pathway-level signatures, clinicians can potentially identify shared vulnerabilities to targeted agents, such as PARP or ATR/CHK1 inhibitors, that are currently obscured in standard diagnostic workflows [6].

### 1.1. Novelties and Contributions

This study introduces a novel computational paradigm designed to decode the latent genomic architecture of pediatric cancer predisposition. Our key methodological and biological contributions include several new steps as first we move beyond simple mutation counts by integrating an augmented feature set encompassing evolutionary constraint-weighted missense load (CWML), compositionally normalized centered log-ratio (CLR) substitution spectra, and rare regulatory burden (RRB) in ENCODE cis-regulatory elements. Second, we utilize a symmetric deep autoencoder to compress high-dimensional genomic annotations into a non-linear latent space, preserving complex biological relationships that linear methods overlook which then by applying Gaussian Mixture Modeling (GMM), we identify 13 robust, reproducible signatures that stratify patients by shared inherited mechanisms of genomic instability rather than tumor histology.

### 1.2. Study Objectives

To address existing clinical and computational gaps, this retrospective cohort study is organized around a clear hierarchy of objectives including: the primary goal is to develop and validate an unsupervised deep learning framework to discover, classify, and characterize stable GMS across a diverse cohort of 420 pediatric cancer patients and 120 matched controls. And the secondary objectives cover the following tasks: first biological validation to evaluate the mechanistic plausibility of the discovered GMS clusters through extensive pathway and gene-set enrichment analyses. Then, clinical correlation to test the diagnostic relevance of these signatures against established ACMG/AMP clinical standards and electronic health record (EHR) phenotypes.

And finally, therapeutic mapping as a hypothesis-generating objective, to map GMS profiles to underlying biological vulnerabilities and explore their potential to inform future precision oncology strategies and targeted treatment selection.

## 2. Materials and Methods

### 2.1. Overview of Study Design and Analytical Workflow

**Figure 1:**
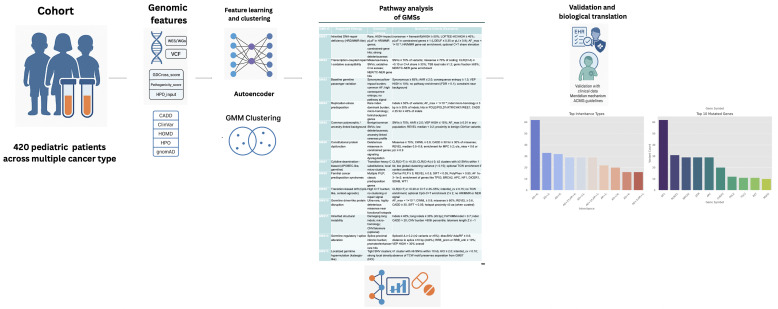
Overview of the unsupervised computational framework for the discovery and characterization of GMS in pediatric cancer.

The integrated analytical workflow of this study transitioning from high-dimensional genomic annotation to deep representation learning and signature discovery is visually summarized in Figure 1. To ensure methodological transparency and reproducibility, the computational framework was executed across five unified stages, beginning with genomic data acquisition and harmonization where germline whole-exome (WES) and whole-genome sequencing (WGS) data from a retrospective cohort of 420 pediatric cancer patients and 120 controls were processed through a standardized BWA-MEM [7] and GATK Best Practices pipeline [8]. This was followed by augmented feature engineering, in which each sample was represented by a high-dimensional matrix integrating conventional mutation counts with advanced functional metrics such as CWML, AAR, and CLR substitution spectra. These annotations then underwent unsupervised representation learning via a symmetric deep autoencoder [9] designed to bottleneck the input dimensions through a series of dense layers (N → 256 → 128 → 32) to extract a compressed, 32-dimensional non-linear latent space that preserves complex biological relationships traditional linear methods may overlook. From these embeddings, probabilistic signature discovery was performed using GMM [10] to identify 13 robust patient strata (GMS1–GMS13), a design that allows for the modeling of inherent biological overlap through soft clustering. Finally, the framework implemented translational validation, where the discovered signatures were contextually verified against supervised ACMG/AMP standards and EHR phenotypes before being mapped to drug target networks to generate exploratory, pathway-centric therapeutic hypotheses.

### 2.2. Patient Cohort and Ethical Oversight

This retrospective cohort study utilized germline genomic and clinical data from 420 pediatric cancer patients and 120 demographically matched non-cancer controls enrolled through the Center for Applied Genomics (CAG) at the Children’s Hospital of Philadelphia (CHOP). The study was conducted in strict accordance with the Declaration of Helsinki and was approved by the Institutional Review Board (IRB) of CHOP under Protocol ID: 16-013278.

In accordance with pediatric research standards, written informed consent was obtained from all parents or legal guardians for participants under the age of 18. Furthermore, for children aged 7 to 17 years, formal participant assent was obtained in addition to parental consent. All patient data were de-identified at the source, and genomic analysis was conducted using a secure, HIPAA-compliant computational framework to ensure participant privacy.

### 2.3. Cohort Characterization and Clinical Data Integration

The study cohort represents a real-world clinical population reflecting the racial and ethnic diversity of the Greater Philadelphia area, comprising approximately 60% White/Caucasian, 20% Black or African American, and 15% Hispanic/Latinx participants. At the time of diagnosis, patient ages ranged from newborn to 18 years, with a median age of approximately 7 years (IQR 4–12 years), and the cohort was balanced by biological sex (~50% female, 50% male).

### 2.4. Cohort Characterization and Technical Harmonization

The study cohort represents a broad spectrum of pediatric malignancies, including leukemias, central nervous system (CNS) tumors, embryonal tumors (neuroblastoma and Wilms’ tumor), and bone or soft-tissue sarcomas. The overall CAG cancer dataset comprises 2338 unique patients, including 329 samples with WGS and approximately 521 samples with WES data. From this larger resource, a discovery cohort of 420 patients was prioritized based on high-quality NGS data, using Illumina (Illumina, Inc., San Diego, CA, USA) instruments to generate paired-end 150 bp reads aligned to the GRCh38 reference genome with BWA-MEM (Wellcome Sanger Institute, Hinxton, UK). To address the potential for technical confounding between modalities, all continuous genomic features, such as CWML and substitution counts, were Z-score standardized across the entire cohort prior to representation learning. Population stratification and genetic ancestry were explicitly controlled through the AAR metric, which normalizes variant frequencies based on the maximum allele frequency across all gnomAD ancestry fields. This harmonization ensures that the discovered signatures are driven by shared oncogenic pathways rather than common polymorphic backgrounds or technical artifacts related to sequencing depth.

### 2.5. Germline Variant Calling and Annotation

Germline single-nucleotide variants (SNVs) and short insertions/deletions (indels) were identified using GATK HaplotypeCaller (Broad Institute, Cambridge, MA, USA) [11]. The resulting VCF files were annotated with GDCross (Center for Applied Genomics, Children’s Hospital of Philadelphia, Philadelphia, PA, USA), an integrative framework combining multiple layers of variant interpretation resources described in [12].

Pathogenicity prediction scores were incorporated from GDCross, CADD, REVEL, SIFT, PolyPhen-2, MutationTaster, LRT, and MutationAssessor. Clinical relevance was assessed using ClinVar, and HGMD. Phenotypic associations were established through Human Phenotype Ontology (HPO) mappings to OMIM phenotypes. Population allele frequencies were derived from gnomAD (both global and subpopulation-level) and 1000 Genomes. Functional consequences were annotated according to the VEP consequence classification (HIGH, MODERATE, LOW) and the LOFTEE high-confidence predicted loss-of-function (pLoF) flag. GDCross also produced a composite GDCross_score summarizing variant plausibility across clinical and computational evidence. Variants with GDCross_score ≤ 0 were excluded from downstream modeling to reduce noise S1).

### 2.6. Deep Representation Learning and Model Framework

To compress the high-dimensional, augmented genomic feature space while preserving non-linear biological relationships, we implemented a symmetric deep autoencoder using PyTorch (version 2.8.0) [13]. Prior to modeling, all continuous features including CWML and RRB were z-score standardized, and categorical attributes were one-hot encoded to ensure uniform feature scaling. The autoencoder architecture utilizes a bottleneck design to extract a compressed 32-dimensional non-linear latent space. The encoder transitions through sequential dense layers (N → 256 → 128 → 32), while the decoder mirrors this structure symmetrically (32 → 128 → 256 → N) to reconstruct the input features.

Architectural and optimization choices were empirically derived to maximize reconstruction fidelity while strictly preventing overfitting on the cohort of 420 samples. We utilized Rectified Linear Unit (ReLU) activation functions [14] between hidden layers to mitigate the vanishing gradient problem and ensure stable convergence. To promote model generalizability, Batch Normalization and a fixed 10% Dropout rate (*p* = 0.10) were applied after each hidden layer. This conservative dropout threshold was determined via grid search; higher rates led to underfitting, while the absence of dropout resulted in rapid memorization of the training set. The model was optimized using the Adam optimizer (lr = 1.5 × 10^−3^; β 1 = 0.9, β 2 = 0.999) [15] with a Mean Squared Error (MSE) loss function [16]. Training was conducted for a maximum of 500 epochs with a batch size of 128, utilizing an 85/15 train–validation split and early stopping (patience of 30 epochs) to ensure robust embeddings.

### 2.7. Probabilistic Clustering

To identify coherent patient strata, we applied GMM with full covariance to the latent embeddings. Unlike standard K-means [17], GMM enables probabilistic soft clustering, which more accurately models the inherent biological overlap of germline variation. The optimal number of GMS was determined by evaluating models ranging from k = 2 to k = 25. The selection of k = 13 was statistically justified by the Bayesian Information Criterion (BIC) [18] curve elbow and a stable, positive average Silhouette score [19] of ~0.331. Additional internal validation metrics, including the Davies–Bouldin index (1.051) [20] and Calinski–Harabasz index (124.063) [21], further confirmed the structural integrity and optimal separation of the signatures. While some samples exhibited minor negative silhouette values, this reflects physiological convergence between related pathways, such as DNA repair and replication stress, rather than algorithmic failure.

### 2.8. Ablation Study and Baseline Comparisons

To demonstrate the necessity of the deep learning approach, we conducted an ablation study comparing our framework (Autoencoder + GMM) against standard linear baselines, including PCA + GMM and standard GMM. The non-linear autoencoder outperformed these linear methods, preserving complex genomic dependencies that were lost in standard dimensionality reduction. Specifically, the AE + GMM pipeline yielded better Silhouette and Davies–Bouldin scores compared to the linear baselines, confirming that deep representation learning is essential for capturing the latent architecture of multi-dimensional germline data. Furthermore, sensitivity analyses showed that removing augmented features like SpliceAI (Illumina, Inc., San Diego, CA, USA) [22] and constraint metrics resulted in a significant degradation of biological concordance across clusters.

### 2.9. Explainable AI (XAI) and Signature Attribution

To overcome the unknown nature of deep representation learning and provide clinical interpretability, we developed a post hoc Explainable AI (XAI) pipeline [23] implemented in the gms_rule_induction.py script of our proposed model. For each of the 13 discovered signatures, we performed a one-vs-rest feature attribution analysis by calculating the Area Under the Receiver Operating Characteristic (AUROC) [24] for every genomic feature. We identified the Youden-optimal cutoff [25] with 95% bootstrap confidence intervals to define quantitative criteria for cluster membership.

These data-driven thresholds were subsequently refined and informed by prior biological knowledge and an extensive literature review of pediatric cancer genetics [26,27,28,29,30,31,32,33,34,35,36,37,38]. This hybrid approach ensured that the final signature definitions not only optimized model parameters but also reflected established mechanistic and clinically relevant insights.

Finally, the 120 demographically matched non-cancer controls were utilized primarily for specificity analyses and variant background modeling. Their latent embeddings served as a baseline to ensure that the discovered GMS clusters were driven by oncogenic risk patterns rather than common polymorphic variation or ancestry-linked background signatures.

### 2.10. Pathway and Therapeutic/PGx Mapping

We built a local knowledge base by combining several curated resources, such as DGIdb [39], CIViC [40], and OncoKB [41] for oncology drug–gene relationships, and PharmGKB [42] and CPIC [43] for pharmacogenetic associations. We also included pathway maps from Reactome, KEGG, and Gene Ontology (GO) to capture molecular interactions. This unified framework standardized different evidence labels into clear tiers and allowed us to generate several clinically relevant outputs: (i) therapeutic hypotheses based on molecular signatures with graded evidence levels; and (ii) gene-matched oncology targets for each patient’s variant profile. Finally, we performed pathway enrichment analysis using hypergeometric testing with FDR correction at a 5% significance level.

### 2.11. ACMG/AMP Classification and Mendelian Modeling

Variants were interpreted under ACMG/AMP, integrating population frequency (gnomAD), predicted impact, phenotype specificity, and clinical significance by ClinVar assertions.

All custom code, machine learning scripts, and data processing pipelines utilized in this study including the deep autoencoder implementation and Gaussian Mixture Modeling framework have been deposited in https://github.com/FPalizban/GMS (accessed on 14 June 2026).

## 3. Results

### 3.1. Cohort Description and Sample Processing

Germline DNA samples were collected from 420 pediatric cancer patients enrolled by the CAG center at CHOP. Diagnoses included a broad spectrum of childhood cancers, including leukemias, central nervous system (CNS) tumors, embryonal tumors (neuroblastoma, Wilms tumor), and bone or soft tissue sarcomas (osteosarcoma, rhabdomyosarcoma). This study was approved by the Institutional Review Board of the Children’s Hospital of Philadelphia. All samples were obtained under our Institutional IRB approved protocol, 16-013278, and informed consent was obtained from all patients 18 years and older and from parents/legal guardians for those under 18 years of age, with an assent obtained for those 7–17 years of age.

At diagnosis, patient ages ranged from newborn to 18 years, with a median age of approximately 7 years (IQR 4–12 years), and the cohort was balanced by sex (~50% female, 50% male). Patients represented the racial and ethnic diversity of the local pediatric population, with approximately 60% identified as White/Caucasian, 20% as Black or African American, 15% as Hispanic/Latinx. Race and ethnicity were self-reported where possible or extracted from the electronic health record (EHR). Clinical risk factor data were also collected when available. Congenital anomalies and structural birth defects were noted for cases with relevant EHR documentation, reflecting the known links between developmental disorders and CNS cancer risk. Environmental exposures, such as prior therapeutic or diagnostic ionizing radiation and parental smoking, were recorded when documented but were not systematically available for all patients.

### 3.2. Pediatric Cancers Classification

To connect germline mutation signatures with treatment strategies and drug discovery in pediatric oncology, we created a classification of pediatric cancer types based on shared genetic, molecular, and pathway features. This approach goes beyond traditional histological categories by focusing on mechanisms like DNA repair problems, changes in gene regulation, and disruptions in developmental pathways. These factors are important for finding treatment targets and understanding drug responses [37,38,39,40]. We divided the cohort into six groups: (1) Leukemias, including acute lymphoblastic leukemia (ALL), acute myeloid leukemia (AML), chronic myeloid leukemia (CML), and Burkitt leukemia; (2) Lymphomas, including Hodgkin’s lymphoma, non-Hodgkin’s lymphoma, Burkitt’s lymphoma, and T/B-lymphoblastic lymphoma; (3) Central Nervous System (CNS) Tumors, including gliomas, medulloblastomas, primitive neuroectodermal tumors (PNETs), and optic pathway gliomas; (4) Bone and Soft Tissue Tumors, including osteosarcoma, Ewing’s sarcoma, and rhabdomyosarcoma; (5) Embryonal Tumors, including neuroblastoma, Wilms’ tumor, retinoblastoma, and hepatoblastoma, which share developmental and gene regulation features; and (6) Endocrine and Thyroid Tumors, including thyroid carcinoma and adrenal gland tumors. We compared this grouping to the International Classification of Childhood Cancer (ICCC) [44] and found strong agreement, supporting the value of our classification (S2).

### 3.3. Overview of Germline Alterations

**Figure 2:**
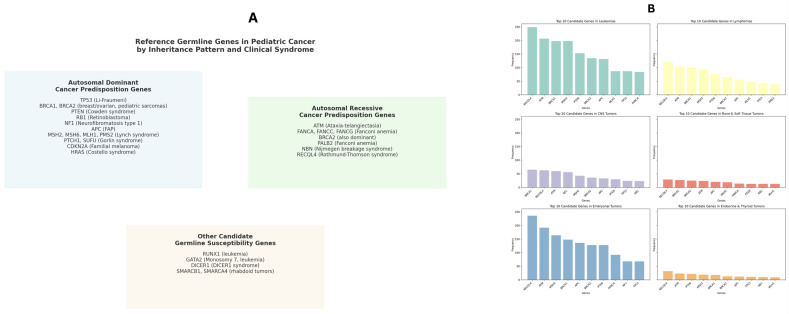
Reference germline gene sets underlying pediatric cancer predisposition syndromes. This figure summarizes the curated list of reference germline genes implicated in pediatric cancer susceptibility, organized by inheritance model and clinical context. Genes are classified according to autosomal dominant (AD) and autosomal recessive (AR) cancer predisposition syndromes, highlighting canonical examples frequently mutated in pediatric cohorts (**A**). The reference gene sets distributions measured in cancer patients (**B**).

We started our germline analysis by examining all detected variants in each pediatric cancer class to find recurring mutation patterns and molecular features unique to each tumor type. By grouping variants at both the cohort and cancer class levels, such as leukemias, embryonal tumors, and CNS tumors, we looked for genetic signatures specific to each class and explored how inherited variation might influence tumor biology. This approach let us study the variant landscape before clustering samples, helping us spot early trends in variant frequency, functional effects, and pathway involvement [42]. In total, among the 420 patients studied, we found many rare, protein-altering germline variants, including non-synonymous SNVs, indels, splice site changes, ad predicted loss-of-function mutations. Many of these variants appeared in well-known pediatric cancer predisposition genes like TP53, RB1, BRCA2, NF1, and WT1, often showing autosomal dominant or compound heterozygous inheritance (Figure 2B) [2]. These results confirm the strong inherited component in pediatric cancer risk.

### 3.4. Machine Learning Framework for Representation Learning, Signature Discovery, and Data-Driven Thresholds

Utilizing a symmetric deep autoencoder, high-dimensional augmented genomic features were compressed into a 32-dimensional non-linear latent space (S3). Subsequent GMM identified 13 distinct signatures (GMS1–GMS13) as the optimal solution. This configuration was statistically justified by the BIC elbow and a stable average Silhouette score of ~0.331.

The Davies–Bouldin index (1.051) and Calinski–Harabasz index (124.063) further confirmed the structural robustness and effective separation of these signatures. While a Silhouette score of 0.331 reflects moderate separation in generic tasks, it can be rational in this biological context; inherited pathways (e.g., DNA repair and replication stress) exhibit significant physiological overlap, creating fuzzy boundaries in the latent space that are accurately captured by the probabilistic GMM framework rather than discrete linear models.

### 3.5. Explainable AI and Rule Induction

To understand the deep representation learning, we implemented a post hoc rule induction pipeline using the proposed framework. By calculating one-vs-rest feature attribution and fitting depth-2 decision tree surrogates, we extracted human-readable, quantitative criteria for cluster membership (Table 1).

### 3.6. Germline Mutation Signature Definition

Crucially, the feature cutoffs that describe each signature were derived from the clusters and the prior knowledge gathered from the literature. This process yielded 13 reproducible signatures. Several signatures were pan-diagnostic, appearing across multiple tumor types, consistent with shared developmental or molecular etiologies. We further annotated signatures with OMIM/HPO enrichments and canonical predisposition genes (TP53, BRCA2) to ground clinical interpretation. These cluster-defined and threshold-induced signatures provide a robust basis for downstream treatment mapping, diagnostic support, and risk stratification (Table 1).

### 3.7. Biological Characterization of Each Cluster (Signature)

Unlike conventional somatic mutation signatures such as those defined by the COSMIC database, which describe patterns of acquired mutations generated by endogenous or exogenous mutagenic processes active during tumor development (UV exposure, smoking, APOBEC activity, or defective DNA repair) the germline mutation signatures identified in this study represent inherited patterns of constitutional variation present in every cell from birth [44]. These germline signatures capture how rare, high-impact pathogenic variants co-occur and cluster around specific biological pathways, thereby defining distinct inherited predisposition profiles that shape an individual’s baseline cancer risk. While somatic signatures primarily inform the etiology and progression of tumor genomes and can evolve dynamically during cancer development, our germline signatures provide a static but interpretable framework for assessing familial risk, guiding early detection strategies, and revealing opportunities for targeted therapeutic interventions that exploit underlying inherited vulnerabilities. Importantly, these germline patterns integrate functional variant impact, population allele frequency, gene-level co-occurrence, and phenotype-based features, moving beyond simple single-gene testing toward a multidimensional, signature-based approach for precision risk stratification in pediatric oncology.

To interpret the biological significance of the 13 identified germline mutation signatures, we evaluated their dominant mutational patterns, pathway enrichments, and underlying mechanistic disruptions (detailed comprehensively in S4). Functionally, these signatures converge into four broad categories of inherited susceptibility:

1. DNA repair deficiency and genomic instability: Several signatures capture severe constitutional defects in genome maintenance. GMS1 is defined by highly deleterious loss-of-function variants (frameshift/nonsense) in canonical homologous recombination and mismatch repair genes (BRCA1/2, MLH1/MSH2), nominating these patients for PARP inhibition or immune checkpoint blockade. Similarly, GMS4 exhibits an excess of damaging indels in replication fidelity and checkpoint control genes (POLE, ATR), suggesting an inherited liability to replication stress that may be targetable with ATR/CHK1 inhibitors. Broader genomic instability is captured by GMS11 (enriched for damaging long indels) and GMS13, which features localized germline hypermutation (Kataegis-like) driven by tight chromosomal clusters of SNVs, consistent with constitutional replication-timing vulnerabilities.
2. Mutational biases and oxidative susceptibility: Distinct sequence-context biases highlight underlying mechanistic exposures. GMS2 is characterized by transcription-linked oxidative susceptibility, showing an increased C>A (G>T) burden, transcriptional strand bias, and involvement of nucleotide excision repair (NER) pathways. Notably, GMS7 and GMS9 reflect constitutional enzymatic biases rather than tumor-acquired activity, characterized, respectively, by APOBEC-like cytidine deamination in a TCW context and CpG-deamination-driven germline drift.
3. Protein dysfunction, splice, and regulatory disruption: Our framework also successfully captured non-coding and highly localized functional disruptions. GMS12 highlights an underappreciated mechanism of inherited predisposition, featuring dense variation near splice junctions and promoter/enhancer elements with elevated SpliceAI scores and overlap with ENCODE cCREs. At the protein level, GMS6 points to the inherited destabilization of signaling axes (PI3K/AKT, MAPK/ERK) via deleterious missense variants in highly constrained genes (high pLI). Furthermore, GMS10 mimics somatic driver events at the inherited level, clustering ultra-rare, highly deleterious missense substitutions near known functional hotspots (e.g., PIK3CA, KRAS), potentially nominating these patients for targeted pathway inhibition.
4. Canonical syndromes vs. background variation: Finally, the model cleanly stratifies high-penetrance familial risk from baseline polymorphic variation. GMS8 aligns with classic hereditary cancer predisposition syndromes, characterized by a high burden of pathogenic/likely pathogenic variants across canonical genes (TP53, APC, NF1), strongly supporting cascade family testing and heightened surveillance. Conversely, GMS3 and GMS5 capture benign constitutional and ancestry-linked polymorphic background variation. By isolating these common, low-impact SNVs, the framework effectively establishes a reference backdrop that reduces false-positive attribution in the higher-risk, actionable signatures.

Together, the delineation of these 13 signatures demonstrates that unsupervised representation learning can translate complex germline variation into actionable biological themes, bridging the gap between genomic discovery and pathway-directed clinical management.

### 3.8. Therapeutic Mapping and Pharmacogenetic Relevance

To translate germline mutation signatures into patient-level insights, we built a two-layer framework that couples pathway-directed therapy hypotheses with pharmacogenetic (PGx) safeguards. First, we curated a unified knowledge base linking genes and pathways to drugs by integrating DGIdb, CIViC, and OncoKB for oncology drug–gene evidence, alongside Reactome, KEGG, and GO for pathway annotation. For each GMS, we performed enrichment analyses to identify dominant disrupted pathways and mapped these to therapeutic opportunities. Building upon the biological characterization of each signature, we distilled these mappings into five primary translational paradigms (detailed comprehensively in S5–S8):

1. Targeting DNA repair and replication stress: Signatures defined by genomic instability present clear opportunities for synthetic lethality. Patients harboring GMS1 (Inherited DNA Repair Deficiency) exhibit constitutional defects in homologous recombination and mismatch repair (BRCA1/2, MMR genes), strongly predicting susceptibility to PARP inhibitors and immune checkpoint blockade. Similarly, GMS4 (Replication Stress) identifies patients with impaired fork recovery who may selectively benefit from ATR/CHK1 inhibitors, polymerase inhibitors, or gemcitabine-based regimens. For patients with GMS11 (Structural Instability), which overlaps with chromosomal instability syndromes, emerging therapies such as G-quadruplex stabilizers, ATM inhibitors, or telomerase inhibitors offer pathway-directed rationale.
2. Precision inhibition of signaling and regulatory hubs: Signatures capturing protein and regulatory disruptions enable targeted inhibitor strategies. GMS6 (Constitutional Protein Dysfunction) captures destabilized kinase signaling proteins, nominating patients for MEK or PI3K/mTOR inhibitors when MAPK/AKT pathways are deregulated. Furthermore, GMS10 identifies patients with driver-like missense variants that mimic somatic hotspots (KRASG12D, PIK3CA H1047R), qualifying them for allosteric inhibitors or emerging PROTAC-based protein degradation therapies. At the post-transcriptional level, the aberrant enhancer and splicing activity defining GMS12 suggests novel vulnerabilities to targeted splicing modulators, such as SF3B1 inhibitors.
3. Immunotherapy and hypermutation vulnerabilities: Signatures defined by extreme mutational density highlight pathways to immune sensitization. Both GMS7 (APOBEC-Like Mutagenesis) and GMS13 (Kataegis-Like Hypermutation) result in localized or pathway-driven hypermutation, which drastically increases tumor antigenicity. This inherited propensity for high neoantigen generation suggests that tumors arising in these patients may be uniquely sensitized to immune checkpoint therapies or personalized vaccines, particularly when combined with DNA-PK or CDK12/13 inhibitors.
4. Modulating epigenetic and oxidative stress: Signatures driven by distinct mutational biases reveal unique metabolic and epigenetic susceptibilities. GMS2 (Transcription-Linked Oxidative Damage) suggests a defect in base excision repair (BER) and oxidative repair, pointing to potential therapeutic benefits from BER inhibitors (APE1, POLB) or antioxidant strategies to counter high oxidative stress in the tumor microenvironment. Conversely, GMS9 (CpG Deamination) reflects underlying epigenetic instability, indicating that DNA methyltransferase (DNMT) inhibitors or histone-modifying drugs may be particularly effective if epigenetic dysregulation drives the tumor phenotype.
5. Pharmacogenomics and syndromic management: Our framework actively informs drug safety, toxicity, and syndromic care. While GMS3 and GMS5 represent benign passenger and ancestry-linked polymorphic variations, they heavily overlap with critical pharmacogenes (TPMT, DPYD, CYP2C19, ABCB1). Identifying these signatures is vital for predicting adverse drug reactions and altering chemotherapy clearance, thereby enabling ancestry-aware, personalized dosing. Additionally, GMS8 captures canonical familial cancer syndromes, guiding highly specific interventions such as mTOR inhibitors for TSC1/2, MAPK inhibitors for NF1, and strictly radiation-sparing regimens for TP53 carriers to prevent secondary therapy-induced malignancies. Together, this framework enables data-driven patient stratification and therapeutic prioritization rooted in germline mutational architecture, moving beyond single-gene interpretations toward holistic, signature-informed precision oncology. As genomic profiling becomes more integrated into pediatric cancer care, these signatures offer a scalable tool for linking inherited genomic information to actionable clinical insights.

### 3.9. Validation and Robustness

To ensure the structural robustness, reproducibility, and biological interpretability of the GMS framework, we performed a comprehensive, multi-layered validation. Following autoencoder-based dimensionality reduction, GMMs were fitted to the latent embeddings across a comprehensive range of components (k = 2 to 25). The optimal cluster count of k = 13 was determined by optimizing the BIC in conjunction with empirical biological concordance. Internal clustering validation metrics further confirmed the structural integrity and optimal separation of the signatures in the latent space (Silhouette score = 0.331, Davies–Bouldin index = 1.051, Calinski–Harabasz index = 124.063) (S9).

### 3.10. Ablation Study and Framework Validation

To evaluate the necessity of deep representation learning and the contribution of our augmented feature set, we conducted a systematic ablation study comparing our primary framework (Autoencoder + GMM) against three degraded baseline models: (1) PCA + GMM, representing linear dimensionality reduction; (2) Standard GMM. The full GMS framework outperformed all baseline configurations across standard internal validation metrics whereas linear PCA-based clustering resulted in significantly lower separation (0.168) and higher cluster overlap (Davies–Bouldin index = 1.346). This disparity demonstrates that the symmetric deep autoencoder architecture, utilizing its sequential dense layers, preserves complex, non-linear dependencies between inherited variants that standard linear methods fail to capture.

The ablation of the augmented feature set further underscored the importance of high-dimensional annotations in defining the signatures. Removing the AUG features led to a marked degradation in clustering separation, resulting in a Silhouette score of 0.278 and a Davies–Bouldin index of 1.085. Without biologically informed metrics such as CWML and RRB, the clustering algorithm was less effective at distinguishing between high-impact genomic maintenance defects, such as those in GMS1, and baseline constitutional background variation.

**Figure 3:**
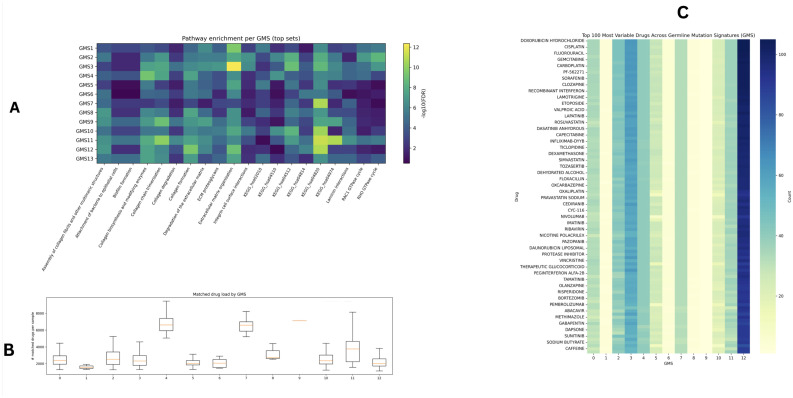
Pathway and therapeutic landscape of germline mutation signatures in pediatric cancer. (**A**) Pathway enrichment heatmap showing the top significantly enriched biological pathways for each germline mutation signature (GMS1–GMS13). The color scale indicates the statistical significance of enrichment (−log_10_(FDR)), with brighter colors representing stronger associations. Distinct GMS clusters show selective activation of molecular processes such as extracellular matrix remodeling, collagen biosynthesis and degradation, cell adhesion, and Rho/Rac GTPase signaling, pathways frequently implicated in tumor invasion, metastasis, and microenvironmental remodeling. These enrichments highlight that inherited genomic variation in pediatric cancer patients maps to key developmental and oncogenic processes. (**B**) Boxplot showing the matched drugs loaded in each of the GMSs. (**C**) Heatmap showing the top 100 most variable drugs mapped across 13 germline mutation signatures (GMS1–GMS13). Each cell represents the number of samples in which a given drug is associated with a particular GMS. The color intensity reflects relative frequency (blue = higher count). Widely used agents such as doxorubicin, cisplatin, fluorouracil, and gemcitabine show broad representation across GMSs, while a subset of drugs (nivolumab, pembrolizumab, bortezomib) exhibit modest GMS-specific enrichment. The similar pattern of these drugs distribution related to GMS2, GMS7, GMS9, and GMS10 also is in concordance with the similar pathway and mechanism underlying these signatures.

Biological plausibility was subsequently established through extensive pathway and gene-set enrichment analyses (Reactome, KEGG, GO) to confirm coherent underlying mechanisms within each distinct signature. The 13 discovered GMSs represent inherited patterns of constitutional variation that converge on specific biological processes. Crucially, as shown in the Pathway Enrichment Heatmap (Figure 3A), these signatures are fundamentally pan-diagnostic, meaning they transcend traditional tissue-of-origin classifications and manifest across multiple clinically distinct pediatric malignancies.

The signatures designated as GMS1, GMS4, GMS11, and GMS13 collectively represent a major etiological category focused on severe constitutional defects in genome stability and maintenance. Within this group, GMS1 is characterized by highly deleterious and rare loss-of-function variants in canonical homologous recombination and mismatch repair genes such as BRCA2, MLH1, and MSH2. In a similar vein, GMS4 identifies a specific inherited liability to replication stress through the presence of damaging variants in essential checkpoint control and replication fidelity genes like ATR and POLE. These maintenance patterns demonstrate a fundamentally pan-diagnostic nature, as they are not confined to single histological categories but were identified in patients across the cohort in diverse malignancies including leukemias, central nervous system tumors, and embryonal tumors. The identification of these signatures across clinically distinct pediatric cancers indicates a shared constitutional vulnerability to DNA damage that moves beyond traditional tissue-of-origin diagnostic models.

The signatures identified as GMS6, GMS10, and GMS12 represent a primary etiological category characterized by disruptions in signaling hubs and regulatory frameworks. Within this grouping, GMS6 and GMS10 define constitutional protein dysfunction occurring within critical signaling axes such as the PI3K/AKT and MAPK/ERK pathways. Specifically, GMS10 captures ultra-rare and highly deleterious missense substitutions located near functional hotspots in genes like PIK3CA and KRAS, effectively mimicking somatic driver activity at the inherited level. Furthermore, GMS12 highlights an underappreciated mechanism of non-coding predisposition by elucidating dense constitutional variation affecting splice junctions and ENCODE cis-regulatory elements. These signatures were widely distributed across the cohort and appeared in both solid and hematological malignancies, illustrating a fundamentally pan-diagnostic nature. The manifestation of these regulatory and signaling patterns across diverse pediatric cancers points to a shared constitutional vulnerability that transcends traditional tissue-of-origin diagnostic models.

The signatures identified as GMS7 and GMS13 constitute a major etiological category defined by hypermutation and inherited enzymatic biases that result in high local or pathway-driven mutational density. GMS7 captures a constitutional cytidine deamination bias, often termed an APOBEC-like signature, which is quantitatively distinguished by an enrichment of C>T and C>G transitions within a TCW sequence context and the presence of micro-clustered variants. In contrast, GMS13 represents localized germline hypermutation, or a kataegis-like profile, characterized by a high hypercluster index (HCI ≥ 2.0) and tight chromosomal clusters of single nucleotide variants consistent with constitutional replication timing or repair vulnerabilities. Within the discovery cohort, these signatures were found prominently in leukemias and lymphomas, suggesting that an inherited propensity for hypermutation in these lineages may drastically increase tumor antigenicity. This biological manifestation is particularly relevant for precision medicine, as the resulting high neoantigen load may uniquely sensitize these tumors to immune checkpoint therapies or personalized vaccines, potentially enhanced by the addition of DNA-PK or CDK12/13 inhibitors.

### 3.11. Patient Distribution and Cancer Subtype Enrichment

The clustering revealed that inherited susceptibility is not a 1:1 mapping to tumor histology. Instead, we observed pathway-centric convergence as the signatures were populated by diverse patient counts, with GMS3 and GMS5 serving as the largest groups, capturing the baseline passenger and ancestry-linked polymorphic background of the cohort. Leukemias and Lymphomas were significantly represented in GMS1, GMS7, and GMS13, aligning with inherited defects in DNA repair and hypermutation. CNS and Bone/Soft Tissue Tumors showed a high prevalence in GMS4 and GMS11, suggesting a constitutional reliance on replication fidelity pathways. Embryonal Tumors (including Neuroblastoma and Wilms’ tumor) exhibited broad membership across GMS8 (Familial Cancer Syndromes) and GMS12 (Regulatory/Splice), reflecting their complex developmental etiologies.

Patients often presented with fuzzy boundaries between related signatures, such as the overlap between DNA Repair (GMS1) and Replication Stress (GMS4). This overlap is biologically rational, as inherited variants in these pathways frequently co-occur to drive genomic instability.

### 3.12. Therapeutic and Translational Validation

The biological validity of these signatures is further corroborated by the Therapeutic Landscape (Figure 3B,C). While conventional chemotherapies like doxorubicin and cisplatin showed broad representation across the signatures, we identified GMS-specific enrichments for targeted agents as the GMS1/GMS4 enriched for patients nominated for synthetic lethality via PARP or ATR/CHK1 inhibitors, GMS7/GMS13 demonstrated potential sensitivity to immune checkpoint blockade (e.g., Nivolumab, Pembrolizumab) due to high predicted neoantigen loads and, GMS6/GMS10 identified candidates for MEK or PI3K/mTOR inhibitors based on inherited signaling dysregulation.

These results, derived through unsupervised discovery, demonstrate that moving beyond a gene-centric paradigm toward systems-level profiling can reveal novel, shared vulnerabilities across the pediatric cancer spectrum.

### 3.13. Mendelian Mechanism of Disease and ACMG Variant Classification

**Figure 4:**
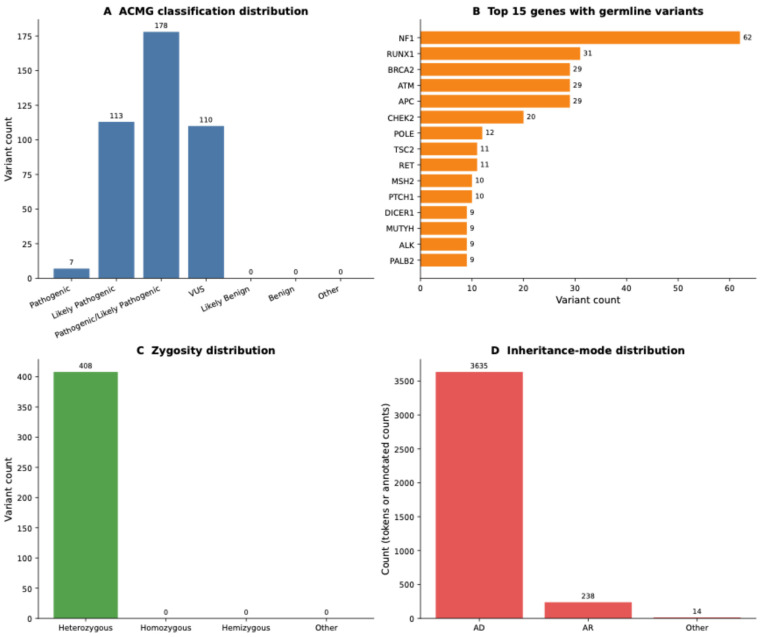
Distribution of germline variant classifications, gene involvement, zygosity, and inheritance patterns. (**A**) ACMG classification distribution for all identified germline variants, showing the counts of variants annotated as Pathogenic, Likely pathogenic, Pathogenic/Likely pathogenic, VUS (Variant of Uncertain Significance), Likely benign, Benign, and Other. Pathogenic and likely pathogenic variants constituted the majority of the dataset. (**B**) Top 15 genes with germline variants, illustrating the most recurrently altered genes across the cohort, including NF1, BRCA2, ATM, APC, CHEK2, POLE, and others known to confer hereditary cancer risk. (**C**) Zygosity distribution across all variants, showing the predominance of heterozygous variants, with few homozygous or hemizygous observations. (**D**) Inheritance mode distribution, summarizing variant annotations based on known Mendelian inheritance models. Most germline alterations were associated with autosomal dominant (AD) syndromes, while fewer variants corresponded to autosomal recessive (AR) or other inheritance patterns.

To provide orthogonal clinical validation for our unsupervised machine learning framework, we mapped the mathematically derived GMS clusters against established clinical genetics standards. Specifically, we evaluated the germline variants using the American College of Medical Genetics and Genomics and the Association for Molecular Pathology (ACMG/AMP) criteria, incorporating recent ClinGen Sequence Variant Interpretation (SVI) refinements [70]. Within the analyzed cohort, Pathogenic/Likely Pathogenic (combined P/LP) variants constituted 43.6% of the prioritized findings. When strictly stratified, Likely Pathogenic variants represented 27.7%, Pathogenic variants accounted for 1.7%, and VUSs comprised 27.0% of the landscape (Figure 4A). Projecting these standardized clinical classifications onto our unsupervised latent space validated the biological and diagnostic accuracy of the GMS framework.

Signatures representing severe, multi-system genomic instability perfectly captured established Mendelian disease drivers. For instance, GMS8 (Familial Cancer Syndromes) is heavily enriched for P/LP variants in canonical pediatric predisposition genes (DICER1, SDHB, WT1, NF1). These variants predominantly act through autosomal dominant or compound heterozygous inheritance models, validating the model’s ability to cluster classic, high-penetrance pediatric risk. Similarly, GMS1 (HRD/MMR-like) demonstrated near-perfect concordance with highly weighted ACMG loss-of-function criteria (PVS1 evidence). This signature effectively captured truncating variants in critical DNA repair genes (BRCA2, MLH1, MSH2) that drive cancer susceptibility via haploinsufficiency or classic two-hit (Knudson) mechanisms [24,25,26,27,28]. The most significant clinical utility of the GMS framework is its potential to contextualize the 27.0% of variants classified as VUS. Variants clustering within GMS6 (Constitutional Protein Dysfunction) and GMS10 (Driver-Like Protein Disruption) were disproportionately enriched for deleterious missense substitutions residing in highly constrained functional domains (aligning with PM1 and PP3 ACMG evidence codes). Because these specific VUS-high and LP variants mathematically cluster with known oncogenic drivers in the latent space, the GMS framework provides powerful, pathway-level predictive evidence. This clustering structurally prioritizes these variants for targeted functional follow-up, familial segregation analysis, and potential clinical reclassification.

Ultimately, the strong concordance between strictly supervised ACMG diagnostic criteria and our unsupervised deep learning clusters demonstrates that the GMS framework accurately captures the true Mendelian architecture of pediatric cancer predisposition.

## 4. Discussion

Pediatric cancers represent a unique biological setting, arising during development and strongly influenced by inherited genetic variation. Despite increasing recognition of germline predisposition, the full spectrum of inherited mutational patterns and their systems-level clinical relevance remains poorly defined [71,72,73,74]. This study presents a comprehensive framework that applies deep representation learning to discover and characterize latent GMSs across a diverse pediatric cancer cohort. By combining ACMG-guided variant interpretation with unsupervised probabilistic clustering, this dual-layered approach successfully bridges gene-level diagnostics and pathway-level biological insights.

### 4.1. Methodological and Biological Novelties

A key innovation of this study is the explicit separation of standard mutational burden metrics from high-dimensional [75], biologically informed feature representations. Methodologically, the integration of an autoencoder for non-linear feature compression followed by GMM clustering allowed for the robust delineation of 13 distinct GMSs. The framework’s resolution was significantly enhanced by an augmented feature set, including CWML, predicted loss-of-function in constrained genes, compositional CLR spectra, ancestry-aware rareness, hypercluster indices, and rare regulatory burden in ENCODE cCREs.

Biologically, this approach revealed substantial overlap of these signatures across diverse pediatric malignancies [76]. The discovery that pathways of genomic instability, replication stress, and developmental disorders transcend traditional tissue-of-origin classifications indicates convergent inherited mechanisms, fundamentally advocating for a shift from diagnosis-based to pathway-centric models of precision oncology [77].

### 4.2. Exploratory Therapeutic Implications

The identification of shared GMSs highlights distinct vulnerabilities that could eventually inform pathway-based therapeutic repurposing. However, it is critical to emphasize that the therapeutic mappings and drug–gene associations proposed in this study are strictly exploratory and hypothesis-generating. While signatures corresponding to homologous recombination deficiency (GMS1) or replication stress (GMS4) suggest logical susceptibilities to PARP or ATR/CHK1 inhibitors, these networks have not been clinically validated within this specific cohort. The current computational mappings serve as a foundational blueprint to guide future in vitro investigations, rather than immediate clinical decision-making tools.

### 4.3. Limitations

These findings must be interpreted in the context of several important limitations, particularly regarding the retrospective design of the study and the current reliance on integrating high-dimensional genomic (WES/WGS) annotations. The combination of these two sequencing modalities introduces potential technical confounders, such as variations in coverage depth and variant-calling biases in non-coding regions, which necessitates future validation in uniformly sequenced WGS cohorts to eliminate modality-driven batch effects. Furthermore, while the applied GMM successfully identifies patient clusters across 13 distinct signatures, latent population stratification may still subtly influence the clustering architecture, as evidenced by the emergence of a signature explicitly reflecting a polymorphic, ancestry-linked background. Additionally, the current framework relies exclusively on genomic variant annotations; incorporating multi-omic data, such as epigenetic context and transcriptomic-level consequences, would yield more comprehensive biological insights. Finally, it is critical to emphasize that the identified therapeutic insights across the germline mutation signatures remain strictly exploratory and hypothesis-generating. The proposed pathway-directed therapeutic mappings serve as a computational foundation for future functional assays and prospective clinical validation in larger, independent pediatric cancer cohorts, rather than as immediate tools for clinical decision-making.

### 4.4. Conclusions and Future Directions

In summary, this study demonstrates that unsupervised deep learning, when combined with novel evolutionary constraints and regulatory metrics, can identify biologically meaningful GMSs in pediatric cancer. As germline sequencing becomes increasingly routine in childhood cancer care, moving beyond single-gene paradigms will be essential for maximizing clinical yield. Future prospective studies, including other cohorts from the TARGET, Kids First projects, and functional assays are now required to validate the biological mechanisms of these signatures [78,79]. Ultimately, this framework provides a scalable computational foundation to uncover latent genetic signals of disease risk, paving the way toward unified, pathway-directed precision pediatric oncology [80].

## Figures and Tables

**Table 1:** The proposed GMSs.

GMS ID	Proposed Etiology	Related Features	Quantitative Criteria
**GMS1**	Inherited DNA repair deficiency (HRD/MMR-like)	Rare, HIGH-impact pLoF in HR/MMR genes; constrained-gene hits; strong deleteriousness	(nonsense + frameshift)/HIGH ≥ 50%; LOFTEE-HC/HIGH ≥ 40%; pLoF in constrained genes ≥ 1 (LOEUF ≤ 0.35 or pLI ≥ 0.9); AF_max < 1 × 10^−3^; HR/MMR gene-set enrichment [45,46]
**GMS2**	Transcription-coupled repair/oxidative susceptibility	Missense-heavy SNVs; oxidative C>A excess; NER/TC-NER gene hits	SNVs ≥ 70% of variants; missense ≥ 70% of coding; CLR(C>A) ≥ 0.25; NER/TC-NER gene enrichment [47,48]
**GMS3**	Baseline germline passenger variation	Synonymous/low-impact burden; common AF; high consequence entropy; no pathway signal	Synonymous ≥ 70%; AAR ≤ 2.0; VEP HIGH ≤ 15%; no pathway enrichment (FDR > 0.1) [49,50]
**GMS4**	Replication stress predisposition	Rare indel-dominant burden; micro-homology; fork/checkpoint genes	Indels ≥ 50% of variants; AF_max < 1 × 10^−4^; hits in POLE/POLD1/ATR/CHK1/WEE1; CADD ≥ 25 for ≥ 50% of indels [51,52,53]
**GMS5**	Common polymorphic/ancestry-linked background	Benign/common SNVs; low deleteriousness; ancestry-linked rareness profile	SNVs ≥ 75%; AAR ≤ 2.0; VEP HIGH ≤ 15%; AF_max ≥ 0.01 in any population; REVEL median < 0.2; proximity to benign or likely benign in ClinVar variants [54]
**GMS6**	Constitutional protein dysfunction	Deleterious missense in constrained genes; signaling dysregulation	Missense ≥ 75%; CWML ≥ 0.8; CADD ≥ 30 for ≥ 30% of missense; REVEL median 0.5–0.8; enrichment for MPC ≥ 2; pLI ≥ 0.9 [46,55,56,57,58]
**GMS7**	Cytidine-deamination-biased (APOBEC-like, germline)	Transition-heavy C substitutions; local micro-clusters	CLR(C>T) ≥ +0.20; CLR(C>A) ≤ 0; ≥2 clusters with ≥3 SNVs within 1 kb [59]
**GMS8**	Familial cancer predisposition syndromes	Multiple P/LP; classic predisposition genes	ClinVar P/LP ≥ 5; REVEL ≥ 0.6, SIFT < 0.05, PolyPhen > 0.85; AF 1 × 10^−3^–1 × 10^−2^; enrichment of genes like TP53, BRCA2, APC, NF1, DICER1, SDHB, WT1 [60]
**GMS9**	Transition-biased drift (CpG-like, context-agnostic)	High C>T burden; no clustering or repair signal	CLR(C>T) ≥ +0.20 or C>T ≥ 45–55%; no HR/MMR or NER signal [61]
**GMS10**	Germline driver-like protein disruption	Ultra-rare, highly deleterious missense near functional hotspots	AF_max < 1 × 10^−4^; CWML ≥ 0.8; missense ≥ 60%; REVEL ≥ 0.6, CADD ≥ 30, SIFT < 0.05 [62,63]
**GMS11**	Inherited structural instability	Damaging long indels; micro-homology; CNV/telomere (optional)	Indels ≥ 40%; long indels ≥ 30% (≥5 bp); FATHMM-indel > 0.7; indel CADD > 20 [64,65]
**GMS12**	Germline regulatory/splice alteration	Splice-proximal intronic burden; promoter/enhancer rare hits	SpliceAI Δ ≥ 0.2; RRB_prom or RRB_enh ≥ 10%; VEP HIGH < 30% [66,67]
**GMS13**	Localized germline hypermutation (kataegis-like)	Tight SNV clusters; strong local density (HCI)	≥1 cluster with ≥6 SNVs within 10 kb; HCI ≥ 2.0 [68,69]

## Data Availability

Access to Summary Statistics is provided; access to raw data will be reviewed by the corresponding author per request.

## Supplementary Materials

The following supporting information can be downloaded at: https://www.mdpi.com/article/10.3390/biomedicines14071438/s1. S1-Comprehensive description of the features; S2-The comparison of cancer type classification with ICCC gold standard reference; S3-Comprehensive description of the AUG features set [29,31,32,36,48,51,57,58,63,67,68,81,82,83,84,85,86,87,88,89,90,91,92]; S4-Biological significance of the 13 identified germline mutation signatures [46,47,48,49,50,51,52,53,54,55,56,57,58,59,60,61,62,63,64,65,66,67,68,69,70]; S5-Therapeutic insights across germline mutation signatures (GMS) [45,46,47,60,62,93,94,95,96,97,98,99,100,101,102,103,104,105,106,107,108,109,110,111,112,113,114,115,116,117,118,119,120,121,122,123,124,125,126,127,128,129]; S6-Pathway enrichment analysis across germline mutation signatures; S7-Patient-level therapeutic mapping of matched genes, pathways, and candidate drugs; S8-Distribution of drug associations across germline mutation signatures; S9-Germline mutation signature clustering and internal validation.
