# Supplementary material for: Unsupervised Deep Representation Learning and Probabilistic Clustering for the Systems-Level Discovery of Germline Mutation Signatures in Pediatric Cancers
Source: Biomedicines. 2026 Jun 24;14(7):1438. doi: 10.3390/biomedicines14071438 (PMC13404483; doi:10.3390/biomedicines14071438)
Supplement: Supplementary file 1 [file biomedicines-14-01438-s001.zip › S3.pdf]

Deleteriousness and Consequence:

Core features included CADD_phred scores (median, count of variants ≥20), REVEL (median), SIFT and PolyPhen-2 summaries, VEP consequence categories (HIGH/MODERATE/LOW), LOFTEE high-confidence predicted loss-of-function (pLoF) variant counts, long-indel flags (≥5 bp), and counts of frameshift and stop-gain variants. To enhance resolution, we introduced constraint-weighted missense load (CWML), calculated by weighting REVEL or AlphaMissense × (CADD/30) by gene constraint (1/LOEUF) and summing across missense variants per sample [1-9].

Splicing and Regulatory Signals:

Splicing-related features included SpliceAI Δ scores (maximum and burden of $\Delta \geq 0.2$), counts of intronic and synonymous variants near canonical splice sites, and distance to splice junctions. Regulatory burden was quantified via overlap with ENCODE candidate cis-regulatory elements (cCREs), partitioned into rare regulatory burden over promoter-like (RRB_prom) and enhancer-like (RRB_enh) elements for variants with AF $<10^{-5}$. Splice density normalized counts of splice-altering variants was included to capture overall splice disruption per sample [10-14].

Population and Constraint Metrics:

Variants were binned by gnomAD allele frequencies (AF $<10^{-4}$, $<10^{-3}$, $\geq 10^{-2}$), with sample-level summaries for minimum, median, and maximum AF values. ClinVar pathogenic/likely pathogenic variant counts were included. Gene-level constraint metrics such as pLI [15-18].

Substitution Spectrum and Mutational Architecture:

SNV classes were standardized to a pyrimidine reference (C>T, C>G, C>A, T>C, T>G, T>A) to form a 6-channel substitution profile [19].

Rareness and Evolutionary Pressure:

We computed Ancestry-Aware Rareness (AAR) as the median $-\log_{10}(\text{max\_AF})$ across all available ancestry-specific and global frequency fields, providing a rareness-adjusted signal that accounts for population stratification and founder effects [20,21].

Pathway and Phenotypic Priors:

Each sample was annotated with membership in curated pathway gene sets, including homologous recombination (HR), mismatch repair (MMR), replication stress, and transcription-coupled repair (Reactome/KEGG).

All continuous numeric features were standardized using z-score normalization across the cohort; categorical variables were one-hot encoded. This high-dimensional feature matrix served as the input for downstream deep learning and clustering models to define GMS subgroups [22,23].

References

[1] Kircher, M., Witten, D. M., Jain, P., O'roak, B. J., Cooper, G. M., & Shendure, J. (2014). A general framework for estimating the relative pathogenicity of human genetic variants. *Nature genetics*, *46*(3), 310-315.

[2] Ioannidis, N. M., Rothstein, J. H., Pejaver, V., Middha, S., McDonnell, S. K., Baheti, S., ... & Sieh, W. (2016). REVEL: an ensemble method for predicting the pathogenicity of rare missense variants. The American Journal of Human Genetics, 99(4), 877-885.

[3] Ng, P. C., & Henikoff, S. (2003). SIFT: Predicting amino acid changes that affect protein function. *Nucleic acids research*, *31*(13), 3812-3814.

[4] Adzhubei, I. A., Schmidt, S., Peshkin, L., Ramensky, V. E., Gerasimova, A., Bork, P., ... & Sunyaev, S. R. (2010). A method and server for predicting damaging missense mutations. Nature methods, 7(4), 248-249.

[5] Karczewski, K. J., Francioli, L. C., Tiao, G., Cummings, B. B., Alföldi, J., Wang, Q., ... & MacArthur, D. G. (2020). The mutational constraint spectrum quantified from variation in 141,456 humans. *Nature*, *581*(7809), 434-443.

[6] Samocha, K. E., Kosmicki, J. A., Karczewski, K. J., O'Donnell-Luria, A. H., Pierce-Hoffman, E., MacArthur, D. G., ... & Daly, M. J. (2017). Regional missense constraint improves variant deleteriousness prediction. *BioRxiv*, 148353.

[7] Cannon, S., Williams, M., Gunning, A. C., & Wright, C. F. (2023). Evaluation of in silico pathogenicity prediction tools for the classification of small in-frame indels. BMC medical genomics, 16(1), 36.

[8] Genes, P. C. (2004). A Systematic Survey of Loss-of-Function Variants in Human. Science, 304, 1663.

[9] Cheng, J., Novati, G., Pan, J., Bycroft, C., Žemgulytė, A., Applebaum, T., ... & Avsec, Ž. (2023). Accurate proteome-wide missense variant effect prediction with AlphaMissense. Science, 381(6664), eadg7492.

[10] Jaganathan, K., Panagiotopoulou, S. K., McRae, J. F., Darbandi, S. F., Knowles, D., Li, Y. I., ... & Farh, K. K. H. (2019). Predicting splicing from primary sequence with deep learning. *Cell*, *176*(3), 535-548.

[11] Cummings, B. B., Marshall, J. L., Tukiainen, T., Lek, M., Donkervoort, S., Foley, A. R., ... & MacArthur, D. G. (2017). Improving genetic diagnosis in Mendelian disease with transcriptome sequencing. *Science translational medicine*, *9*(386), eaal5209.

[12] Anna, A., & Monika, G. (2018). Splicing mutations in human genetic disorders: examples, detection, and confirmation. Journal of applied genetics, 59(3), 253-268.

[13] Moore, J. E., Purcaro, M. J., Pratt, H. E., Epstein, C. B., Shoresh, N., Adrian, J., ... & Weng, Z. (2020). Expanded encyclopaedias of DNA elements in the human and mouse genomes. *Nature*, *583*(7818), 699-710.

[14] ENCODE Project Consortium. (2012). An integrated encyclopedia of DNA elements in the human genome. *Nature*, *489*(7414), 57.

[15] Khurana, E., Fu, Y., Colonna, V., Mu, X. J., Kang, H. M., Lappalainen, T., ... & Gerstein, M. (2013). Integrative annotation of variants from 1092 humans: application to cancer genomics. *Science*, *342*(6154), 1235587.

[16] GTEx Consortium. (2020). The GTEx Consortium atlas of genetic regulatory effects across human tissues. Science, 369(6509), 1318-1330.

[17] Lek, M., Karczewski, K. J., Minikel, E. V., Samocha, K. E., Banks, E., Fennell, T., ... & Exome Aggregation Consortium. (2016). Analysis of protein-coding genetic variation in 60,706 humans. Nature, 536(7616), 285-291.

[18] Landrum, M. J., Lee, J. M., Benson, M., Brown, G. R., Chao, C., Chitipiralla, S., ... & Maglott, D. R. (2018). ClinVar: improving access to variant interpretations and supporting evidence. Nucleic acids research, 46(D1), D1062-D1067.

[19] Alexandrov, L. B., Nik-Zainal, S., Wedge, D. C., Aparicio, S. A., Behjati, S., Biankin, A. V., ... & Stratton, M. R. (2013). Signatures of mutational processes in human cancer. *nature*, *500*(7463), 415-421.

[20] Sirugo, G., Williams, S. M., & Tishkoff, S. A. (2019). The missing diversity in human genetic studies. Cell, 177(1), 26-31.

[21] Mathieson, I., & McVean, G. (2014). Demography and the age of rare variants. *PLoS genetics*, *10*(8), e1004528.

[22] Kanehisa, M., & Goto, S. (2000). KEGG: kyoto encyclopedia of genes and genomes. Nucleic acids research, 28(1), 27-30.

[23] Draghici, S., Khatri, P., Tarca, A. L., Amin, K., Done, A., Voichita, C., ... & Romero, R. (2007). A systems biology approach for pathway level analysis. Genome research, 17(10), 1537-1545.
