# Supplementary material for: Unsupervised Deep Representation Learning and Probabilistic Clustering for the Systems-Level Discovery of Germline Mutation Signatures in Pediatric Cancers
Source: Biomedicines. 2026 Jun 24;14(7):1438. doi: 10.3390/biomedicines14071438 (PMC13404483; doi:10.3390/biomedicines14071438)
Supplement: Supplementary file 1 [file biomedicines-14-01438-s001.zip › S9.pdf]

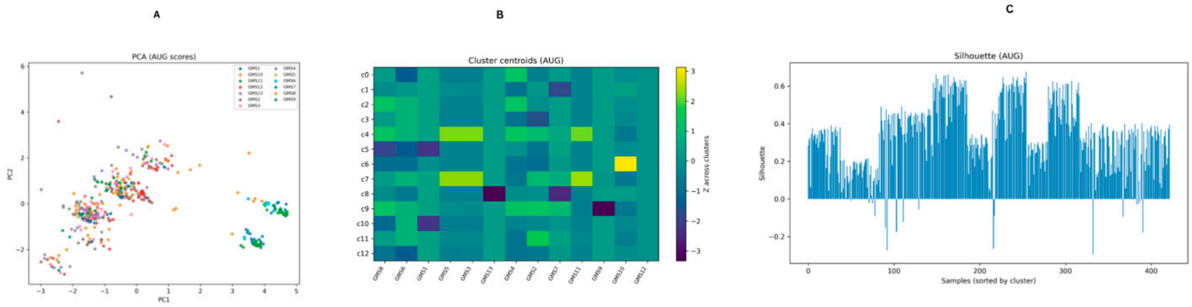

Germline mutation signature (GMS) clustering and validation. (A) Principal component analysis (PCA) of the feature-encoded germline dataset demonstrates clear separation of samples into multiple GMS clusters, with partial overlap reflecting shared biological pathways across cancer types. (B) Cluster centroids for the 13 GMSs showing differential enrichment across key feature dimensions. Distinct patterns correspond to predominant etiologic mechanisms, while moderate cross-cluster similarity reflects biological convergence between repair- and replication-associated pathways. (C) Silhouette coefficient distribution for the Gaussian mixture model (GMM) clustering. The average silhouette score is positive ~0.331, indicating overall well-defined clustering structure. Although a small subset of samples exhibits slightly negative silhouette values, this is expected given the biological overlap of germline mutation signatures across distinct pediatric cancer types, where shared inherited pathways (e.g., DNA repair, replication stress) prevent complete discrimination of subgroups.
