# Supplementary material for: Unsupervised Deep Representation Learning and Probabilistic Clustering for the Systems-Level Discovery of Germline Mutation Signatures in Pediatric Cancers
Source: Biomedicines. 2026 Jun 24;14(7):1438. doi: 10.3390/biomedicines14071438 (PMC13404483; doi:10.3390/biomedicines14071438)
Supplement: Supplementary file 1 [file biomedicines-14-01438-s001.zip › S5.pdf]

Therapeutic insights across germline mutation signatures (GMS)

GMS ID (Etiology)	Therapeutic Insights
GMS1 – DNA-Repair Deficiency (HRD/MMR-like)	Patients with inherited DNA-repair defects (BRCA1/2, MMR genes) may respond to PARP inhibitors and immune checkpoint blockers. ATR, CHK1, or WEE1 inhibitors can further exploit replication stress, and dual checkpoint [1-5].
GMS2 – Transcription-Linked Oxidative Damage	Defects in NER and oxidative repair pathways suggest potential benefit from BER inhibitors (APE1, POLB) or antioxidants like N-acetylcysteine to counter high oxidative stress in tumors [6-8].
GMS3 – Passenger Variation	Though non-pathogenic, this pattern informs pharmacogenomic care. Overlaps with *TPMT*, *DPYD*, or *CYP2C19* variants can predict adverse drug reactions, enabling personalized drug selection and dosing [9-11].
GMS4 – Replication Stress	These patients show impaired DNA replication and fork recovery. ATR/CHK1 inhibitors, polymerase inhibitors, or gemcitabine-based regimens may target replication vulnerability effectively [12-15].
GMS5 – Polymorphic / Ancestry-Linked Background	Reflects benign genetic diversity that influences drug metabolism. Variants in transporter genes (*ABCB1*, *SLCO1B1*) may alter exposure or clearance, emphasizing ancestry-aware pharmacogenomics [16-18].
GMS6 – Protein Dysfunction in Signaling Pathways	Variants disturbing kinase or signaling proteins may respond to MEK inhibitors or PI3K/mTOR inhibitors when MAPK or AKT pathways are deregulated [19-21].
GMS7 – APOBEC-Like Mutagenesis	High mutation density and neoantigen generation from APOBEC activity may sensitize tumors to vaccines, or immune checkpoint therapies, particularly in hypermutated leukemias and lymphomas [22, 23].
GMS8 – Familial Cancer Syndromes	Targeted options depend on the gene: mTOR inhibitors for *TSC1/2*, MAPK inhibitors for *NF1*, *WT1*vaccines for relapsed leukemia, and radiation-sparing regimens for *TP53* carriers [24-26].
GMS9 – CpG Deamination / Epigenetic Instability	Suggests DNA methylation fragility. DNMT inhibitors or histone-modifying drugs may help when epigenetic dysregulation is evident in tumors [27, 28].
GMS10 – Driver-Like Missense Variants	Germline mutations mirroring somatic hotspots (*KRAS* G12D, *PIK3CA* H1047R) can qualify patients for allosteric/PROTAC-based inhibitors [29-31].
GMS11 – Structural Instability	Overlaps with chromosomal instability syndromes. G-quadruplex stabilizers, ATR/ATM inhibitors, or telomerase inhibitors may provide benefit in tumors with structural genomic stress [32-35].
GMS12 – Splice / Regulatory Variants	Aberrant splicing or enhancer activity may be targeted with SF3B1 modulators [36-38].
GMS13 – Kataegis-Like Hypermutation	Local hypermutation can increase tumor antigenicity. DNA-PK or CDK12/13 inhibitors, along with checkpoint blockade, may enhance immune response. Genome-stability agents are also potential options [39-42].

References

[1] Lord, C. J., & Ashworth, A. (2017). PARP inhibitors: Synthetic lethality in the clinic. *Science*, *355*(6330), 1152-1158.

[2] Le, D. T., Durham, J. N., Smith, K. N., Wang, H., Bartlett, B. R., Aulakh, L. K., ... & Diaz Jr, L. A. (2017). Mismatch repair deficiency predicts response of solid tumors to PD-1 blockade. *Science*, *357*(6349), 409-413.

[3] Lecona, E., & Fernandez-Capetillo, O. (2018). Targeting ATR in cancer. *Nature Reviews Cancer*, *18*(9), 586-595.

[4] Karnitz, L. M., & Zou, L. (2015). Molecular pathways: targeting ATR in cancer therapy. *Clinical cancer research*, *21*(21), 4780-4785.

[5] Saini, P., Li, Y., & Dobbelstein, M. (2015). Wee1 is required to sustain ATR/Chk1 signaling upon

[6] Helleday, T., Eshtad, S., & Nik-Zainal, S. (2014). Mechanisms underlying mutational signatures in human cancers. Nature reviews genetics, 15(9), 585-598.replicative stress. *Oncotarget*, *6*(15), 13072.

[7] Robertson, A. B., Klungland, A., Rognes, T., & Leiros, I. (2009). DNA repair in mammalian cells: Base excision repair: the long and short of it. *Cellular and molecular life sciences*, *66*(6), 981-993.

[8] Caston, R. A., Gampala, S., Armstrong, L., Messmann, R. A., Fishel, M. L., & Kelley, M. R. (2021). The multifunctional APE1 DNA repair–redox signaling protein as a drug target in human disease. Drug Discovery Today, 26(1), 218-228.

[9] Relling, M. V., & Evans, W. E. (2015). Pharmacogenomics in the clinic. *Nature*, *526*(7573), 343-350.

[10] Amstutz, U., Henricks, L. M., Offer, S. M., Barbarino, J., Schellens, J. H., Swen, J. J., ... & Schwab, M. (2018). Clinical Pharmacogenetics Implementation Consortium (CPIC) guideline for dihydropyrimidine dehydrogenase genotype and fluoropyrimidine dosing: 2017 update. Clinical Pharmacology & Therapeutics, 103(2), 210-216.

[11] Relling, M. V., Schwab, M., Whirl-Carrillo, M., Suarez-Kurtz, G., Pui, C. H., Stein, C. M., ... & Yang, J. J. Clinical Pharmacogenetics Implementation Consortium (CPIC) guideline for thiopurine dosing based on TPMT and NUDT15 genotypes: 2018 update. Clin Pharmacol Ther.

[12] Zeman, M. K., & Cimprich, K. A. (2014). Causes and consequences of replication stress. Nature cell biology, 16(1), 2-9.

[13] Cimprich, K. A., & Cortez, D. (2008). ATR: an essential regulator of genome integrity. Nature reviews Molecular cell biology, 9(8), 616-627.

[14] Liu, S., Opiyo, S. O., Manthey, K., Glanzer, J. G., Ashley, A. K., Amerin, C., ... & Oakley, G. G. (2012). Distinct roles for DNA-PK, ATM and ATR in RPA phosphorylation and checkpoint activation in response to replication stress. Nucleic acids research, 40(21), 10780-10794.

[15] McNeely, S., Conti, C., Sheikh, T., Patel, H., Zabludoff, S., Pommier, Y. G., ... & Tse, A. (2010). Chk1 inhibition after replicative stress activates a double strand break response mediated by ATM and DNA-dependent protein kinase. Cell cycle, 9(5), 995-1004.

[16] Thorn, C. F., Klein, T. E., & Altman, R. B. (2013). PharmGKB: the pharmacogenomics knowledge base. In Pharmacogenomics: Methods and Protocols (pp. 311-320). Totowa, NJ: Humana Press.

[17] Niemi, M. (2010). Transporter pharmacogenetics and statin toxicity. Clinical Pharmacology & Therapeutics, 87(1), 130-133.

[18] Wolking, S., Schaeffeler, E., Lerche, H., Schwab, M., & Nies, A. T. (2015). Impact of genetic polymorphisms of ABCB1 (MDR1, P-glycoprotein) on drug disposition and potential clinical implications: update of the literature. Clinical pharmacokinetics, 54(7), 709-735.

[19] Samatar, A. A., & Poulikakos, P. I. (2014). Targeting RAS–ERK signalling in cancer: promises and challenges. Nature reviews Drug discovery, 13(12), 928-942.

[20] Fruman, D. A., Chiu, H., Hopkins, B. D., Bagrodia, S., Cantley, L. C., & Abraham, R. T. (2017). The PI3K pathway in human disease. Cell, 170(4), 605-635.

[21] LoRusso, P. M. (2016). Inhibition of the PI3K/AKT/mTOR pathway in solid tumors. Journal of clinical oncology, 34(31), 3803-3815.

[22] Roberts, S. A., Lawrence, M. S., Klimczak, L. J., Grimm, S. A., Fargo, D., Stojanov, P., ... & Gordenin, D. A. (2013). An APOBEC cytidine deaminase mutagenesis pattern is widespread in human cancers. Nature genetics, 45(9), 970-976.

[23] Wang, S., Jia, M., He, Z., & Liu, X. S. (2018). APOBEC3B and APOBEC mutational signature as potential predictive markers for immunotherapy response in non-small cell lung cancer. Oncogene, 37(29), 3924-3936.

[24] De Blank, P. M., Gross, A. M., Akshintala, S., Blakeley, J. O., Bollag, G., Cannon, A., ... & Fisher, M. J. (2022). MEK inhibitors for neurofibromatosis type 1 manifestations: Clinical evidence and consensus. Neuro-oncology, 24(11), 1845-1856.

[25] Hirahata, T., Ul Quraish, R., Ul Quraish, A., Ul Quraish, S., & Ul Quraish, R. (2025). WT1 gene: a potential therapeutic target for multiple cancer treatment strategies. Clinical and Experimental Medicine, 25(1), 336.

[26] Frebourg, T., Bajalica Lagercrantz, S., Oliveira, C., Magenheim, R., & Evans, D. G. (2020). Guidelines for the Li–Fraumeni and heritable TP53-related cancer syndromes. European Journal of Human Genetics, 28(10), 1379-1386.

[27] Sved, J., & Bird, A. (1990). The expected equilibrium of the CpG dinucleotide in vertebrate genomes under a mutation model. Proceedings of the National Academy of Sciences, 87(12), 4692-4696.

[28] Jones, P. A., Issa, J. P. J., & Baylin, S. (2016). Targeting the cancer epigenome for therapy. Nature Reviews Genetics, 17(10), 630-641.

[29] Suda, K., Tomizawa, K., & Mitsudomi, T. (2010). Biological and clinical significance of KRAS mutations in lung cancer: an oncogenic driver that contrasts with EGFR mutation. Cancer and Metastasis Reviews, 29(1), 49-60.

[30] Gustin, J. P., Cosgrove, D. P., & Park, B. H. (2008). The PIK3CA gene as a mutated target for cancer therapy. Current cancer drug targets, 8(8), 733-740.

[31] Neklesa, T. K., Winkler, J. D., & Crews, C. M. (2017). Targeted protein degradation by PROTACs. Pharmacology & therapeutics, 174, 138-144.

[32] Andor, N., Maley, C. C., & Ji, H. P. (2017). Genomic instability in cancer: teetering on the limit of tolerance. Cancer research, 77(9), 2179-2185.

[33] Shay, J. W., & Wright, W. E. (2006). Telomerase therapeutics for cancer: challenges and new directions. Nature reviews Drug discovery, 5(7), 577-584.

[34] Zegers, J., Peters, M., & Albada, B. (2023). DNA G-quadruplex-stabilizing metal complexes as anticancer drugs. JBIC Journal of Biological Inorganic Chemistry, 28(2), 117-138.

[35] Weber, A. M., & Ryan, A. J. (2015). ATM and ATR as therapeutic targets in cancer. Pharmacology & therapeutics, 149, 124-138.

[36] Seiler, M., Yoshimi, A., Darman, R., Chan, B., Keaney, G., Thomas, M., ... & Buonamici, S. (2018). H3B-8800, an orally available small-molecule splicing modulator, induces lethality in spliceosome-mutant cancers. Nature medicine, 24(4), 497-504.

[37] Szelest, M., & Giannopoulos, K. (2024). Targeting splicing for hematological malignancies therapy. BMC genomics, 25(1), 1067.

[38] Saez, B., Walter, M. J., & Graubert, T. A. (2017). Splicing factor gene mutations in hematologic malignancies. Blood, The Journal of the American Society of Hematology, 129(10), 1260-1269.

[39] Sakofsky, C. J., Roberts, S. A., Malc, E., Mieczkowski, P. A., Resnick, M. A., Gordenin, D. A., & Malkova, A. (2014). Break-induced replication is a source of mutation clusters underlying kataegis. Cell reports, 7(5), 1640-1648.

[40] Schweizer, M. T., Ha, G., Gulati, R., Brown, L. C., McKay, R. R., Dorff, T., ... & Alva, A. (2020). CDK12-mutated prostate cancer: clinical outcomes with standard therapies and immune checkpoint blockade. JCO precision oncology, 4, 382-392.

[41] Mohiuddin, I. S., & Kang, M. H. (2019). DNA-PK as an emerging therapeutic target in cancer. Frontiers in oncology, 9, 635.

[42] Graham, L. S., Pritchard, C. C., & Schweizer, M. T. (2021). Hypermutation, mismatch repair deficiency, and defining predictors of response to checkpoint blockade. Clinical Cancer Research, 27(24), 6662-6665.
