# Supplementary material for: Unsupervised Deep Representation Learning and Probabilistic Clustering for the Systems-Level Discovery of Germline Mutation Signatures in Pediatric Cancers
Source: Biomedicines. 2026 Jun 24;14(7):1438. doi: 10.3390/biomedicines14071438 (PMC13404483; doi:10.3390/biomedicines14071438)
Supplement: Supplementary file 1 [file biomedicines-14-01438-s001.zip › S4.pdf]

GMS1: Inherited DNA Repair Deficiency (HRD/MMR-like)

This signature is enriched for rare predicted loss-of-function variants (frameshift/nonsense, LOFTEE-HC) with high deleteriousness (CADD ≥20) in homologous recombination and mismatch repair genes, including BRCA1/2, TP53, MLH1, and MSH2 [1]. The profile reflects constitutional defects in DNA repair that predispose to genomic instability and suggests therapeutic susceptibility to PARP inhibition and, in selected contexts, immune checkpoint blockade [2].

GMS2: Transcription-Linked Oxidative Susceptibility

Dominated by missense SNVs with elevated REVEL, this signature shows features consistent with transcription-coupled repair vulnerability and oxidative damage during active transcription, including an increased C>A (G>T) burden, transcriptional strand bias, and involvement of NER genes [3]. Variants frequently map to promoters/enhancers or regulatory elements of oncogenes (KRAS, EGFR), indicating a germline predisposition that may be modulated by ROS-targeting strategies or kinase pathway inhibition [4].

GMS3: Baseline Germline Passenger Variation

Characterized by a predominance of synonymous or low-impact variants at higher allele frequencies and lacking coherent pathway enrichment, this signature reflects benign constitutional background variation [5, 6].

GMS4: Replication-Stress Predisposition

This cluster exhibits an excess of rare indels and damaging variants in genes essential for replication fidelity and checkpoint control (POLE, POLD1, ATR), with high predicted impact [7, 8]. The profile is consistent with inherited replication-stress liability and may nominate candidates for ATR/CHK1-directed strategies in rapidly proliferative pediatric tumors [9].

GMS5: Common Polymorphic / Ancestry-Linked Background

Enriched for common, low-impact SNVs that track with population allele frequencies and ancestry principal components, this signature captures constitutional polymorphic background rather than causal predisposition. It provides a reference backdrop to reduce false-positive attribution in other, high-risk signatures [10].

GMS6: Constitutional Protein Dysfunction

Defined by deleterious missense variants with high CADD [55]and moderate-to-high REVEL [11] or AlphaMissense [12]in constrained genes (high pLI), this signature points to inherited destabilization of protein function across signaling axes such as PI3K/AKT and MAPK/ERK. It suggests a predisposition to pathway dysregulation [13, 14].

GMS7: Cytidine-Deamination Bias (APOBEC-like, Germline)

This signature shows an enrichment of C>T/C>G events in TCW sequence context and micro-clustered variants, consistent with constitutional cytidine-deamination bias rather than tumor-acquired activity [15].

GMS8: Familial Cancer Predisposition Syndromes

Patients harbor multiple pathogenic/likely pathogenic variants (high ClinVar P/LP burden with concordant SIFT/PolyPhen/REVEL) across canonical predisposition genes (TP53, BRCA2, APC, NF1). This signature aligns with classic hereditary cancer syndromes, supporting cascade family testing, heightened surveillance, and consideration of DNA repair-targeted interventions [16].

GMS9: CpG-Deamination–Driven Germline Drift

Marked by a high proportion of C>T transitions at CpG dinucleotides [17].

GMS10: Germline Driver-Like Protein Disruption

This cluster comprises ultra-rare, highly deleterious missense substitutions (SIFT-damaging/PolyPhen-probably damaging/CADD >30) that localize near known functional hotspots (PIK3CA, KRAS). The pattern mimics driver-like effects at the inherited level, nominating individuals for targeted pathway inhibition and, where feasible, protein-degradation strategies [18, 19].

GMS11: Inherited Structural Instability

Enriched for damaging long indels (FATHMM-indel high) [20, 21].

GMS12: Germline Regulatory/Splice Alterations

This signature features dense intronic/synonymous variation near splice junctions and promoter/enhancer elements, with elevated SpliceAI [22] scores and overlap with ENCODE cCREs. It highlights underappreciated noncoding mechanisms of predisposition splicing disruption and regulatory mis-expression that may be addressable with splicing modulators or epigenetic therapies [23].

GMS13: Localized Germline Hypermutation (Kataegis-like)

Defined by tight chromosomal clusters of SNVs (short inter-mutation distances, high clustering skew), this profile is consistent with replication-timing/repair vulnerabilities acting constitutionally [24, 25].

References

[1] Le, D. T., Durham, J. N., Smith, K. N., Wang, H., Bartlett, B. R., Aulakh, L. K., ... & Diaz Jr, L. A. (2017). Mismatch repair deficiency predicts response of solid tumors to PD-1 blockade. *Science*, *357*(6349), 409-413.

[2] Lord, C. J., & Ashworth, A. (2017). PARP inhibitors: Synthetic lethality in the clinic. Science, 355(6330), 1152-1158.

[3] Alexandrov, L. B., Nik-Zainal, S., Wedge, D. C., Aparicio, S. A., Behjati, S., Biankin, A. V., ... & Stratton, M. R. (2013). Signatures of mutational processes in human cancer. *nature*, *500*(7463), 415-421.

[4] Trachootham, D., Alexandre, J., & Huang, P. (2009). Targeting cancer cells by ROS-mediated mechanisms: a radical therapeutic approach?. *Nature reviews Drug discovery*, *8*(7), 579-591.

[5] 1000 Genomes Project Consortium. (2015). A global reference for human genetic variation. Nature, 526(7571), 68.

[6] Lek, M., Karczewski, K. J., Minikel, E. V., Samocha, K. E., Banks, E., Fennell, T., ... & Exome Aggregation Consortium. (2016). Analysis of protein-coding genetic variation in 60,706 humans. *Nature*, *536*(7616), 285-291.

[7] Campbell, B. B., Light, N., Fabrizio, D., Zatzman, M., Fuligni, F., de Borja, R., ... & Shlien, A. (2017). Comprehensive analysis of hypermutation in human cancer. *Cell*, *171*(5), 1042-1056.

[8] Palles, C., Cazier, J. B., Howarth, K. M., Domingo, E., Jones, A. M., Broderick, P., ... & Data Analysis: McVean Gilean (Lead) 2 Donnelly Peter 2 10 Cazier Jean-Baptiste 2 Broxholme John 2 Grocock Russell 7 Hatton Edouard 2 Holmes Christopher C 2 10 Hughes Linda 2 Humburg Peter 2 Kanapin Alexander 2 Lunter Gerton 2 Murray Lisa 7 Rimmer Andy 2. (2013). Germline mutations affecting the proofreading domains of POLE and POLD1 predispose to colorectal adenomas and carcinomas. *Nature genetics*, *45*(2), 136-144.

[9] Buisson, R., Boisvert, J. L., Benes, C. H., & Zou, L. (2015). Distinct but concerted roles of ATR, DNA-PK, and Chk1 in countering replication stress during S phase. *Molecular cell*, *59*(6), 1011-1024.

[10] Bergström, A., McCarthy, S. A., Hui, R., Almarri, M. A., Ayub, Q., Danecek, P., ... & Tyler-Smith, C. (2020). Insights into human genetic variation and population history from 929 diverse genomes. Science, 367(6484), eaay5012.

[11] Rentzsch, P., Witten, D., Cooper, G. M., Shendure, J., & Kircher, M. (2019). CADD: predicting the deleteriousness of variants throughout the human genome. *Nucleic acids research*, *47*(D1), D886-D894.

[12] Ioannidis, N. M., Rothstein, J. H., Pejaver, V., Middha, S., McDonnell, S. K., Baheti, S., ... & Sieh, W. (2016). REVEL: an ensemble method for predicting the pathogenicity of rare missense variants. *The American Journal of Human Genetics*, *99*(4), 877-885.

[13] Cheng, J., Novati, G., Pan, J., Bycroft, C., Žemgulytė, A., Applebaum, T., ... & Avsec, Ž. (2023). Accurate proteome-wide missense variant effect prediction with AlphaMissense. Science, 381(6664), eadg7492.

[14] Tokheim, C. J., Papadopoulos, N., Kinzler, K. W., Vogelstein, B., & Karchin, R. (2016). Evaluating the evaluation of cancer driver genes. *Proceedings of the National Academy of Sciences*, *113*(50), 14330-14335.

[15] Roberts, S. A., Lawrence, M. S., Klimczak, L. J., Grimm, S. A., Fargo, D., Stojanov, P., ... & Gordenin, D. A. (2013). An APOBEC cytidine deaminase mutagenesis pattern is widespread in human cancers. Nature genetics, 45(9), 970-976.

[16] Knapke, S., Nagarajan, R., Correll, J., Kent, D., & Burns, K. (2012). Hereditary cancer risk assessment in a pediatric oncology follow-up clinic. *Pediatric blood & cancer*, *58*(1), 85-89.

[17] Sved, J., & Bird, A. (1990). The expected equilibrium of the CpG dinucleotide in vertebrate genomes under a mutation model. Proceedings of the National Academy of Sciences, 87(12), 4692-4696.

[18] Adzhubei, I. A., Schmidt, S., Peshkin, L., Ramensky, V. E., Gerasimova, A., Bork, P., ... & Sunyaev, S. R. (2010). A method and server for predicting damaging missense mutations. *Nature methods*, *7*(4), 248-249.

[19] Garraway, L. A., & Jänne, P. A. (2012). Circumventing cancer drug resistance in the era of personalized medicine. *Cancer discovery*, *2*(3), 214-226.

[20] Ferlaino, M., Rogers, M. F., Shihab, H. A., Mort, M., Cooper, D. N., Gaunt, T. R., & Campbell, C. (2017). An integrative approach to predicting the functional effects of small indels in non-coding regions of the human genome. BMC bioinformatics, 18(1), 442.

[21] Montgomery, S. B., Goode, D. L., Kvikstad, E., Albers, C. A., Zhang, Z. D., Mu, X. J., ... & 1000 Genomes Project Consortium. (2013). The origin, evolution, and functional impact of short insertion–deletion variants identified in 179 human genomes. Genome research, 23(5), 749-761.

[22] Jaganathan, K., Panagiotopoulou, S. K., McRae, J. F., Darbandi, S. F., Knowles, D., Li, Y. I., ... & Farh, K. K. H. (2019). Predicting splicing from primary sequence with deep learning. *Cell*, *176*(3), 535-548.

[23] Khurana, E., Fu, Y., Colonna, V., Mu, X. J., Kang, H. M., Lappalainen, T., ... & Gerstein, M. (2013). Integrative annotation of variants from 1092 humans: application to cancer genomics. Science, 342(6154), 1235587.

[24] Morganella, S., Alexandrov, L. B., Glodzik, D., Zou, X., Davies, H., Staaf, J., ... & Nik-Zainal, S. (2016). The topography of mutational processes in breast cancer genomes. Nature communications, 7(1), 11383.

[25] Nik-Zainal, S., Alexandrov, L. B., Wedge, D. C., Van Loo, P., Greenman, C. D., Raine, K., ... & Stratton, M. R. (2012). Mutational processes molding the genomes of 21 breast cancers. *Cell*, *149*(5), 979-993.
