# Supplementary material for: Unsupervised Deep Representation Learning and Probabilistic Clustering for the Systems-Level Discovery of Germline Mutation Signatures in Pediatric Cancers
Source: Biomedicines. 2026 Jun 24;14(7):1438. doi: 10.3390/biomedicines14071438 (PMC13404483; doi:10.3390/biomedicines14071438)
Supplement: Supplementary file 1 [file biomedicines-14-01438-s001.zip › S1.pdf]

Comprehensive description of the features

The BASE feature set included conventional summaries of variant burden and consequence counts and fractions of SNVs and indels; missense, nonsynonymous, frameshift and splice categories; VEP impact levels (HIGH, MODERATE, LOW); the fraction of high-confidence pLoF variants among VEP-HIGH calls (LOFTEE-HC); and sample-level counts of ClinVar Pathogenic or Likely Pathogenic variants. Deleteriousness was summarized as the mean of CADD, REVEL, SIFT and PolyPhen scores. Population frequency was captured by the median allele frequency across all available gnomAD population fields. Splice and regulatory impact were summarized using SpliceAI (maximum score). We also included a pyrimidine-standardized six-channel substitution spectrum (C>A, C>G, C>T, T>A, T>C, T>G) with a 0.5 pseudocount to stabilize low-burden samples. Categorical attributes were one-hot encoded and continuous features were z-score standardized prior to modeling.

To increase sensitivity and interpretability, we developed an augmented ("AUG") feature set using GRCh38 reference resources (GENCODE, ENCODE candidate cis-regulatory elements, UCSC CpG islands, gnomAD gene-level constraint metrics, and precomputed SpliceAI scores). These augmented features included: (i) constraint-weighted missense load (CWML), which sums REVEL or AlphaMissense × (CADD/30) for each missense variant weighted by gene constraint (1/LOEUF); (ii) counts of high-impact pLoF variants in constrained genes (LOEUF ≤ 0.35 or pLI ≥ 0.85); (iii) a centered log-ratio transformation of the six-channel substitution spectrum; (iv) ancestry-aware rareness (AAR), defined as the median $-\log_{10}$ of the maximum allele frequency across ancestry-specific fields; (v) a hypercluster index (HCI), defined as the maximum z-score of 10-kb windowed variant counts per chromosome; (vi) rare regulatory burden, the fraction of ultra-rare variants (AF $<10^{-5}$) overlapping ENCODE promoter-like (RRB_prom) or enhancer-like (RRB_enh) cCREs; and (vii) splice density, the count of variants with SpliceAI $\Delta \geq 0.2$ normalized by total variant count.
