# Supplementary material for: Unsupervised Deep Representation Learning and Probabilistic Clustering for the Systems-Level Discovery of Germline Mutation Signatures in Pediatric Cancers
Source: Biomedicines. 2026 Jun 24;14(7):1438. doi: 10.3390/biomedicines14071438 (PMC13404483; doi:10.3390/biomedicines14071438)
Supplement: Supplementary file 1 [file biomedicines-14-01438-s001.zip › S2.pdf]

Table 1. The comparison of cancer type classification with ICCC gold standard reference

**Our Clustering**	**ICCC Mapping**
Leukemias	Matches ICCC Group I
Lymphomas	Matches ICCC Group II
CNS Tumors	Matches ICCC Group III
Bone & Soft Tissue Tumors	Combination of ICCC Groups VIII + IX
Embryonal Tumors	Aggregates ICCC Groups IV (neuroblastoma), V (retinoblastoma), VI (Wilms), VII (hepatoblastoma)
Endocrine & Thyroid	Subset of ICCC Group XI
